# Using staged teaching and assessment approaches to facilitate inter-university collaboration and problem-based learning

**DOI:** 10.3389/fpubh.2024.1334729

**Published:** 2024-03-11

**Authors:** Henry Dawson, Gayle Davis, Kirstin Ross, Marie Vaganay Miller, Alastair Tomlinson

**Affiliations:** ^1^School of Sport and Health Sciences, Cardiff Metropolitan University, Wales, United Kingdom; ^2^College of Science and Engineering, Flinders University, Adelaide, SA, Australia; ^3^Faculty of Computing, Engineering and the Built Environment, Ulster University, Coleraine, United Kingdom

**Keywords:** problem-based learning, collaboration, public health, environmental health, scalable, higher education

## Abstract

This article describes the segmented module design and problem-based learning approaches employed to enable parts of a higher education environmental health module (course) to be shared between universities in Wales, Northern Ireland, and Australia. The module requires students to identify the needs and assets of a community then design community-based interventions to address problems and undertake an evaluation of those interventions. Accreditation body and the degree program learning outcome requirements in the UK and Australia were found to hold many comparable knowledge, skills, and graduate attribute criteria, eliminating a potential barrier for international learning and teaching collaboration between higher education institutions. Instead, barriers to collaboration were associated with institutional issues and practicalities such as timetabling and assessment requirements. Taking a segmented approach to module design allowed staged and varied levels of collaboration between participating institutions, all delivering modules (courses) with similar learning outcomes. This provided a more sustainable environment to facilitate shared learning and teaching and fostered closer relations between programs, within these constraining factors. Students using problem-based learning and its group-working component exhibited the development of leadership, communication, and independent learning skills.

## 1 Background and rationale for the module

This article describes the learning and teaching approaches used on a 40-credit Intervention for Health and Sustainable Development module “the module” taken in the final year of an Environmental Health BSc program at Cardiff Metropolitan University (Wales, UK). Innovative module design and a problem-based learning (PBL) approach enabled parts of the module to be shared with two other universities (Flinders University in Adelaide, Australia and Ulster University in Belfast, Northern Ireland).

Academic terminology varies between institutions and countries. The term “module” is used in this article to describe what other institutions may refer to as a “unit” or “course” and describes a discrete subject-specific area of learning with its own learning outcomes (1) and assessments. Multiple modules would be undertaken in a given academic year. Cardiff Metropolitan University also uses “terms” to describe the teaching periods used to break up the academic year in the same way as semesters are used at other institutions.

The Environmental Health BSc uses a spiral curriculum design (2) with learning outcomes, teaching, and assessment, constructively aligned (1) and mapped against the requirements of the Chartered Institute of Environmental Health (CIEH) UK governing body for environmental health. Complex problems and developing scenarios are key learning tools used for teaching. These are matched closely to or based around real-life situations, sourced through the close ties the delivery team have with the environmental health profession and employers.

The module teaches students to assess and prioritize health needs of a specific population, to develop a detailed environmental health community level intervention on an identified issue, in the context of a community regeneration program. Students learn how to evaluate policy frameworks for environmental health, wellbeing, and sustainable development, and consider the influence that can be exerted by environmental and public health professionals to bring about a positive impact on health.

The module was designed with reference to Cardiff Metropolitan University’s Ethical, Digital, Global and Entrepreneurial (EDGE) graduate attributes and aligned with the CIEH curriculum for accredited degree programs. This curriculum included: Dahlgren and Whitehead’s 1991 conceptualization of the determinants of health and wellbeing (3), assessment, management, and communication of risk, health-based sciences, knowledge acquisition and transfer, development and innovation, evidence-based practice, and dissemination of ideas and information. The module design also considers feedback from employers around the need for university graduates to have experience of working within teams.

To provide an authentic learning experience for students, the authors gave access to a real location with introductory information and links to live public health and population census data sources on the community. The students were encouraged to expand on this through self-driven research using live online information sources relevant to their designated location.

The module at Cardiff Metropolitan University has similar learning outcomes to modules on an MSc program at Flinders University (Adelaide, Australia) and an undergraduate BSc program (final year) at Ulster University (Belfast, Northern Ireland, UK). To provide a more externally focused and internationalized learning experience for students across the three institutions, the module contents were matched between the institutions and locations were provided in each university’s locality, for students across all three programs to undertake scoping of the needs and assets of local communities and design appropriate community level intervention plans to improve population health, in a different geographical area to where they were conducting their studies.

Students across each of the partnering institutions worked in groups within their host institution. Each group was allocated to one of the following locations:

- Maerdy and Ferndale – a post-industrial, semi-rural area in South Wales (population 7,255).
- Great Palm Island – an island off the east coast of Australia (population 2,098).
- Ardoyne – an inner-city area in Belfast (population 5,987).

All three locations have different histories and geography, but the health-related challenges faced by the populations have similar origins in the wider determinants of health for the communities in question. All areas have public health observatory and census data, and local authority web site information on activities and amenities within the locality.

Before this collaboration the Module Leader at Cardiff Metropolitan University had worked closely with Stanford University in the USA and the Modern University for Business and Science in Lebanon, developing processes and practices for virtual student exchange to facilitate collaborative day-to-day groupwork for students, between institutions (4). The intention of this project was to draw on that experience and use a scalable segmented module design to allow various levels of collaboration across multiple environmental health programs. Collaboration could then be scaled, from sharing fixed elements (e.g., the communities focused on), through to students working in small cross-institutional groups on shared projects. This stepped approach would provide staged pathway, making it easier to move to a point of full student collaboration.

Each level of collaboration would have benefits for student learning and would bring academics closer together. Achieving the ideal of cross-institutional small group working has the potential to realize the intercultural and social benefits of close student and staff working. It enables learning about other cultures, networking, forming close bonds with those outside of students’ normal social spheres, and exposes students to the commonalities that exist between different populations, challenging prejudices, and preconceptions. Managing learning and teaching collaboration through technology also provides a more environmentally sustainable and low-cost mechanism for accessing the benefits of physical student and staff exchange through international travel (4).

This article captures the module part way through this development and collaboration journey. At the time of writing all three institutions were sharing the three geographical areas detailed above. Introductory recordings were created by native instructors, describing each of the geographical areas. Lecturers from the Cardiff and Belfast institutions were using Microsoft Teams/Panopto to share lecture content and perform assessed presentations around the scoping of the needs and assets of the communities. Recordings of the assessed student presentations were shared between the universities to allow students to see the presentations of their peers. Teaching staff attended presentations remotely, asking the students questions through Microsoft Teams and participating in grading of student groups.

In all instances, students are introduced to their allocated geographical areas at the beginning of the taught sessions and are initially tasked with undertaking an assessment of health needs within the population having regard to the social determinants of health (3). Students are required to use all their prior learning from their environmental health studies to consider the assets and needs of the community. As students across all programs are allocated geographical areas that may be culturally diverse from those they are used to, the process ensures the students have to extend and expand their perceptions of both environmental health and the role of practitioners in the field.

Once a needs assessment is undertaken, students then apply a problem-based learning methodology to investigate and plan potential interventions to improve their identified health needs in their given population. This may involve an investigation of pollution prevention interventions, housing interventions or a broader public health intervention capable of impacting a wider range of determinants. Students will approach their chosen issue by identifying elements they are unfamiliar with, defining what they need to investigate, researching, synthesizing the knowledge through discussion, and repeating as necessary. This active constructivist approach lends itself to collaborative learning across curricula. The subject matter and activities undertaken mimic public health work carried out by environmental health practitioners in the participating countries.

## 2 Pedagogic frameworks underlying the module

### 2.1 The UK and Australian environments

As part of a broader team, the authors have recently undertaken an exercise mapping the environmental health practice requirements for the UK, the US and Australia. As noted above, environmental health curricula in the UK are aligned to the CIEH national accrediting body. Similarly, in Australia, the professional body, Environmental Health Australia (EHA), has an accreditation skills and knowledge matrix, which all accredited awards must align their course content against. The team mapped CIEH criteria against EHA criteria, and the mapping demonstrated that there is significant overlap in the skills and knowledge acquired by university students undertaking environmental health degree awards in either country. There were few skills that were unique to individual countries. The authors consider that the level of comparability serves to demonstrate that the countries featured in this article (the UK and Australia) can consider allowing graduates to practice as EHPs in either location with little further study (5).

Within the UK, Cardiff Metropolitan University final year students study at Level 6 of the Credit and Qualifications Framework for Wales. This matches Level 6 of the National Qualifications Framework for England and Northern Ireland. The relevant requirements at this level of study are summarized in the Welsh level descriptor as follows:

“Achievement at Level 6 reflects the ability to refine and use relevant understanding, methods and skills to address complex problems that have limited definition. It includes taking responsibility for planning and developing courses of action that are able to underpin substantial change or development, as well as exercising broad autonomy and judgment. It also reflects an understanding of different perspectives, approaches or schools of thought and the theories that underpin them (6).”

Students are required to manage their own learning through primary sources, displaying an appreciation for the uncertainty, ambiguity and limits of knowledge including the use of data which may be incomplete. Students must be able to devise and sustain arguments and solve problems. Students must review, consolidate, extend, and apply their knowledge to carry out projects and communicate information, ideas, problems and solutions to specialist and non-specialist audiences (7). The Welsh level descriptor goes on to require students to evaluate actions, methods and results and their implications, and to exercise broad autonomy of judgment, taking responsibility for the work and roles of others (6).

These requirements are reflected in Cardiff Metropolitan University’s Generic Grade Band Descriptors used to develop marking criteria for student work. Flinders University and Ulster University have similar descriptors which set out the expectations of a student studying at each academic level awarded by the institution. At Cardiff Metropolitan and Ulster Universities program ‘levelness’ is audited through the five-yearly internal periodic review of programs and external benchmarking against equivalents in other institutions, via the annual review of programs, through a system of visiting external examiners. Flinders University has a similar process.

Finally, the CIEH governing body curriculum requires students on accredited programs to be able to establish the nature of hazards and make judgments on risk, provide reports and presentations, and show how the acquisition, assimilation and application of knowledge can be used to generate options for resolution of environmental health-based problems. Specific to public health, student must understand surveillance and assessment of population health and wellbeing and the use of data relating to determinants of health and wellbeing. They must be able to assess the evidence of the effectiveness of interventions, to improve population health and wellbeing. The CIEH emphasizes the need for consideration of collaborative working.

**Figure 1:**
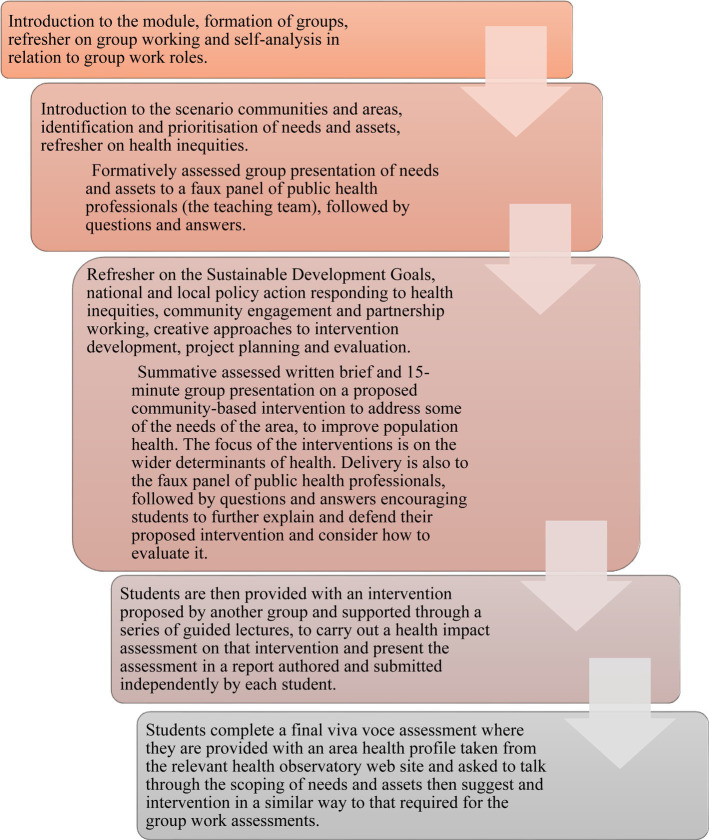
Flow chart showing the student journey through the module.

The module structure, learning outcomes, and learning, teaching and assessment approaches are tailored to reflect the above requirements. Students on the module work in groups of up to six. The module follows a pattern of a two-hour ‘keynote’ session each week, with content tailored to the stages of the journey groups must go through. The module ‘learning journey’ is set out in Figure 1.

### 2.2 Problem-based learning (PBL)

The module uses PBL as a key element of its delivery mechanism. This student-centered approach, first pioneered by McMaster University and Maastricht University in the field of medicine in the 1970’s (8), is a pedagogical system which involves working on authentic problems, in groups, with tutor facilitation (9). Based within the philosophical movement of pragmatism developed by the philosopher and educator John Dewey, PBL is considered an experiential process that is both active in its nature, and involves the activation of prior learning (10). PBL can also be seen to have cognitive constructivist foundations where new knowledge is constructed on a framework of existing knowledge through a process of social interaction (11). Early years educators and founding fathers of constructivist approaches, Vygotsky and Piaget, espouse the provision of challenge in a structured environment to allow learners to achieve maximum learning potential and deep learning (12). Through the developing collaboration on this module with national and international partner organizations this approach seeks to extend the challenge and the potential for deep learning.

The module seeks to use the constructivist learning principles of, learning in context, active learning, and sharing of knowledge generation, by putting students into groups of between four and six, and encouraging them to follow a clear method to meet these principles. This involves clarifying unknown terms, defining the problem, analyzing the problem, sharing learning tasks, research, sharing learning, synthesizing learning, and identifying further research as needed (11). This form of inquiry-based learning using authentic questions and real-world problems has been shown to be effective in activating prior knowledge in the students and providing a framework for further knowledge building (9).

The PBL approach has been shown to improve the quality of the learning process by developing reflective, critical, and collaborative skills in learners (13) while developing and refining leadership skills in those that participate in the process (14). Long-term knowledge retention has been found to improve (15) and there is an enhancement in “deep learning” where students show greater ability to understand content rather than to simply reproduce facts (16). Learners’ inquiry-based learning skills have been seen to improve when engaging with the process (17) as have their skills in communication, problem solving and independent learning (18). In addition to these skills, student satisfaction with the learning process is often greater when PBL is used (18–20).

Although there are positives to the approach, some negatives are also reported in the literature including a reduction in short term knowledge gain due to the more protracted nature of the PBL process (20) and no measurable improvement in “surface learning”; a student’s ability to reproduce facts (16, 17). PBL also requires greater human resources and continuous training to be successful and this investment of time and resources can be viewed as a negative (18).

PBL in this module is facilitated through group work which helps students with the development of new perspectives (21), development of teamworking skills (22) and enhancements in learning (23). This process does need to be managed carefully however as unequal participation, time, and differences in learning speed (24) can all affect the productivity of groups. For the summative assessed groupwork presentations a peer assessment approach was implemented to improve the fairness of grading. The online system allowed group members to confidentially score their peers based on criteria produced by the cohort at the start of term. These scores were used to adjust the group marks to give individual student marks based on their level of contribution to the group’s activities.

It is hoped that groups can also become multi-institutional, to allow students to maximize the positives that can come from group working, as cultural diversity of learning groups has been shown to be especially valuable in developing globalized values and behaviors (25).

## 3 Learning environment

Cardiff Metropolitan University operates a two-term model consisting of two twelve-week teaching blocks between September and early April. The module forms a third of the final-year curriculum for Environmental Health students equating to 40 credits or 400 notional learning hours of study. At Ulster University the module carries 20 credits of their final-year curriculum and adopts most of the content of Cardiff’s module. This is delivered in a smaller number of learning sessions. Delivery methods are closely aligned to those at Cardiff. The delivery methods are further described in Table 1. Learning in the Flinders University version of the module is at master’s level and is also delivered over 20 credits.

**Table 1:** Module delivery methods.

Learning and teaching method	Pedagogical rationale
Scheduled situational learning	Facilitated group work sessions on campus to prepare students to work in groups, run presentations and allow for question-and-answer sessions in formative exercises prior to the assessed presentation for the module.
Scheduled synchronous learning	For the PBL element students are placed into groups for the first half of the module and given weekly keynote lectures on campus. 3 hours are provided per week for Term 1 (Semester 1), but some weeks will have more taught sessions, and some will have more time provided for group work, as the demands of the tasks require. Term 2 (Semester 2) teaches Health Impact Assessment. Taught sessions prepare students for the assessments using a didactive, whole class discussion teaching approach.
Independent guided learning	Group working time is included in the independent study requirements.

At Cardiff and Ulster Universities the module is delivered as a campus-based module, but students are encouraged to participate in their groupwork outside of lesson times as well as during timetabled facilitated PBL sessions (independent guided learning). Students are given access to a channel for each team within a dedicated Microsoft Teams site. This is used as a file exchange and for remote meetings when not in class. Access to good internet and adequate information technology equipment is an essential element of this module and the university has systems in place to ensure students can borrow all necessary equipment and can access the internet on campus throughout the day and evening. This helps to manage the impact of digital poverty on learner engagement. Learning at Flinders University is all online due to the geographical dispersion of students.

Cohort sizes on participating programs are between 20 and 45. The cohorts are mostly ‘home students’, across a range of ages. This module is also shared internally at Cardiff Metropolitan University as an optional module for students on the Food Science and Nutrition BSc.

At Cardiff, three members of staff teach the module, at Ulster University the module is delivered by two staff, and at Flinders University there is one instructor. All instructors delivering the module have experience with PBL delivery.

Ulster University’s module uses the same learning outcomes as Cardiff’s module but omits the emphasis on criticality in learning outcomes 1, 2 and 4.

The learning outcomes for the module are as follows:

1. Critically appraise local, national, and international frameworks for sustainable development, health & wellbeing.
2. Critically assess the evidence on health and wellbeing needs in a defined community, including reference to health inequities, and prioritize issues for intervention.
3. Work in partnership with others to develop a strategic intervention response designed to effect positive changes within an identified community, including a clear strategy for evaluating the effectiveness of the intervention.
4. Critically evaluate the impact of policies, programs and interventions on health and wellbeing.

The first three outcomes relate to the first part of the module where interventions are developed, and the plan is put in place. The fourth learning outcome is taught toward the end of the module where students are allocated an intervention plan from the first half of the module, and they are required to undertake a health impact assessment of their allocated community level intervention.

At Flinders University the learning outcomes are as follows:

1. Demonstrate an understanding of the importance of sustainable development and other relevant concepts and their application in environmental health.
2. Understand the influence of social, economic, cultural and political contexts on environmental health outcomes.
3. Understand the influence of climate change on environmental health and identify ways in which environmental health professionals can advocate remedies to minimize public health impacts.

Subject matter crossover was possible directly or indirectly for all learning outcomes, but operator verbs indicate the different cognitive levels at each institution. This reflects the notional learning hours devoted to the module at each institution. See Table 2.

**Table 2:** Module notional learning hours across the three providers.

	Cardiff Metropolitan	Ulster University	Flinders University
Lectures/tutorial	108	48	4
Self-driven study (including groupwork)	292	152	36
Total hours learning	400	200	40

Indicative content for the Cardiff module included:

1. Sustainable development and public health – international frameworks, national and local action.
2. Determinants of health, health inequities and how to reduce them.
3. Team roles, dynamics, and how effective teams operate.
4. Assessing community needs and assets: identifying and prioritizing issues for intervention.
5. Developing a strategic, creative approach to intervention and identifying key partners and stakeholders.
6. Project planning techniques in intervention development and evaluation.
7. Participation and engagement of local communities in policy development and decision making.
8. Health impact assessment process, practice and reporting.
9. Monitoring and evaluation.

Ulster University covered items 1, 2, 4, 5 and 8. Flinders University covered items 1 and 2 and 5.

**Figure 2:**
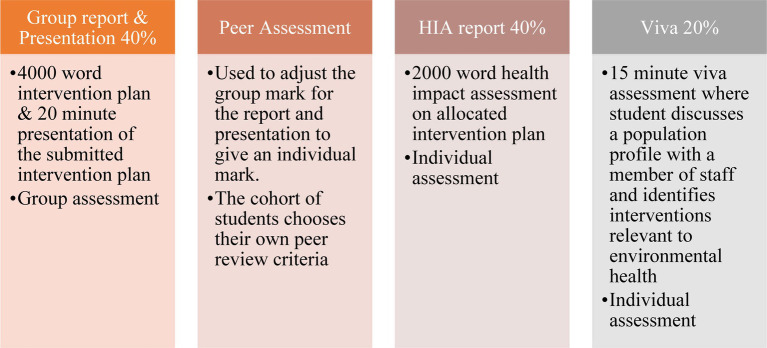
Assessments used on the module.

Cardiff had more contact time so was able to teach to a greater depth and assess more thoroughly, permitting exploration of the subject matter to a higher cognitive level. For Flinders University the opposite was true. Variation in the cognitive level was accommodated through assessment marking rubrics and variation in the learning hours was accommodated through different numbers of summative assessments. The full diet of assessment available for the module is shown in Figure 2.

Due to a lack of module time for formative assessment opportunities in the Ulster variation of the module they used the initial community needs and assets scoping exercise as a five-minute summative assessed group presentation with 5 min of questions from the assessors. Ulster asked students to submit a shorter version of the intervention plan as an individual student report (3,000 words) and to complete an individual health impact assessment using a workshop format, with attendance and participation being graded alongside the 3,000 report (10% of the total mark). The two exercises made up the second assessment for the module. Flinders also retained the presentation assessment with student groups reporting on the scoping exercise and intervention plan in a single assessed presentation of 10 min duration, followed by questions from the assessors.

## 4 Results, practical implications, and lessons learned

To evaluate the impacts of the PBL approach, a deductive thematic analysis was undertaken of student feedback justifying the peer assessment mark they awarded and module evaluation data. Six years of data was analyzed from 2012 to 2020 to establish themes around positives and negatives students experienced when engaging with both PBL and groupwork while studying this module.

### 4.1 Personal development and self-improvement

Improvement of self and the development of skills like leadership, communication and independent learning skills are widely identified by students in their evaluation feedback, there is a recognition that participation in the process can be of benefit even though it may not be comfortable.

‘Student A carried out a lot of individual reading to put into our work, supported the group at times of pressure, and took on other bits of work that may not have been completed by other members. There were some elements of negative thinking and annoyance concerning other members but apart from that student A accepted people’s problems and carried on with everyone to get the tasks done. Student A was quite nervous to present the first task but did it anyway and pulled it off. Student A was also then prepared to contribute to the questioning on the second presentation.’ (Student B, 2017-18)

### 4.2 Reflective skills

The module process facilitates students’ ability to reflect on themselves and their peers through the peer assessment process. Students were able to identify when colleagues had benefitted from self-reflection which had improved their performance.

‘Student C was an excellent group member. She was very proactive, clearly making the work a priority, and offering innovative ideas in group discussions. She recognized her weaknesses early with regards to writing style, and always completed her work very far in advance, to ensure that the rest of the group had plenty of time to read and consider what she had written and make adjustments as necessary.’ (Student D, 2015-16)

### 4.3 Frustrations with the process

Some issues identified by the literature regarding frustrations with group working were apparent in the analysis of the student reasoning. Unequal participation recurred in every year group to varying degrees with differences in learning speed and working practices were also a prominent frustration.

‘Throughout group meetings Student E was sat at their computer, with all of us believing they was carrying out group work, but none was produced. I thought it was a lack of understanding but when asked whether they were clear and understood the task they always said yes, however, two days before the submission they said that they didn’t understand and needed help, at this stage we discovered they had not done any work!’ (Student F, 2015-16)

### 4.4 Students appreciated the reasons for using PBL and its potential benefits

Module feedback indicated that student recognized the challenges in the PBL process. However, they also understood the reasoning which underpinned the approach and appreciated the benefits it could provide.

‘I feel that this module was a very challenging module but in a good way. I feel that having to look at different sets of data for different areas was interesting and then having to think of our own interventions was good. I felt you allowed us to get on with module by ourselves which allowed a more flexible approach to it.’ (Student N, 2022-23)

Module evaluations are not yet available for the Flinders and Ulster University collaborators, but the module has been running in a similar format for over a decade at Cardiff Metropolitan University.

### 4.5 Multi-institutional collaboration and scalability

The authors’ work with the Environmental Health Community of Practice has illustrated the international nature of the teaching undertaken by academics across the world. This work has further enhanced academics’ understanding of the global transferability of teaching and practice in environmental health, by clearly demonstrating that the social determinants of health significantly impact poorer, marginalized people, be they from a remote First Nations island, inner city Belfast, or a rural area. Importantly, the delivery of this module enhanced relationships between the participating academics, providing each other with support, guidance and encouragement. This network of support has resulted in a significantly better teaching resources and approaches than would be developed alone and a more globalized delivery of the subject matter. Collaborative research and publication activities have been initiated by the participating academics, due to the bonds formed through the sharing of their teaching activities.

The segmented approach to the module design allowed collaboration between universities on some points in the module and independent working on others. The range of scenarios could be varied without the need for time-consuming formal modifications to be made, which would be subject to individual institutional regulations. The structure of the module provided a format which could easily be shared with other institutions using common subject matter (learning outcomes). The sharing of the module and inter-institutional interactions between students/staff could be scaled up or down with little interruption to delivery patterns. This sort of scalable ‘supermarket shelf’ approach was found to be necessary for managing the differing levels of collaboration and helped when gradually building closer ties and trust. It provided a more pragmatic, staged approach in the path to full virtual student exchange.

Content was adapted for the Australian postgraduate students and scaled down to present scenarios for their interrogation, with the assessment being reflective, rather than a whole development piece. The most key component for Australian students was the acknowledgment that the social determinants of health, which in Australia are primarily taught using First Nations disadvantage to illustrate principles which affect disadvantaged groups in other wealthy countries. This recognition is important as it gives a global lens through which the typically Australian content is taught.

At Ulster University, the module is adapted and scaled down to fit a 20 credit points modular framework. This was motivated by the opportunities that inter-institutional collaboration provided both to enhance the student and staff experience. The module at Ulster University follows a similar pace, process, and assessment type to Cardiff Metropolitan University for the first weeks of the first term allowing for deeper collaboration be it of shared teaching or shared student group work. The module is currently being delivered for the first time and some constraints, discussed below, have limited this sharing experience this year.

The module evaluation comments made by students at Cardiff Metropolitan University indicated that peer assessment was perceived as a fair way of assessing the intervention reports and presentations. Peer evaluation was brought in after the summative assessment mark had been awarded. Students were not given the opportunity to get feedback on their performance and respond. Sridharan, Tai and Boud (26) note that students may not see themselves as others do. Other authors describe the need for students to have practice at evaluating their peers (27, 28). Introducing a two-step peer assessment process where students evaluated group member performance after the formatively assessed scoping presentation and the summative assessment of the proposed interventions would provide students with an opportunity to change their behavior. This opportunity for poor performers to change was thought to outweigh the potential of this measure to introduce conflict at a point where groups are starting to become more established in their systems, roles, and processes.

## 5 Constraints on the module and delivery

Institutional processes required to modify module learning outcomes, delivery, and assessment to allow closer collaboration between the Cardiff and Ulster universities took a year to complete. This should have permitted full student virtual exchange and shared lecture delivery but timetabling placed module sessions on different days. Timetabling also removed any potential for shared lectures (through Microsoft Teams) with students at Flinders University, due to time zone differences.

Collaboration on module delivery and assessment could be managed as long as some module learning outcomes were matched. Flinders University only hosted a Graduate Diploma level environmental health program so mostly taught and assessed at a higher cognitive level. Both Flinders and Ulster universities also had different numbers of learning credits (notional learning hours) allocated to the modules which had been matched against the Cardiff module.

Embarking on shared teaching and learning requires minimum levels of IT infrastructure and skills for collaborating parties. If lecture theaters are to be linked, then broadband must be strong and both teaching rooms must be small, or have AV equipment with sensitive microphones to pick up student questions. The AV equipment must also have audio channels which can manage interaction over videoconferencing applications such as Microsoft Teams. Instructors managing sessions need to be sufficiently familiar with the hardware and software involved to be able to manage basic troubleshooting as IT support is not immediately available at the time of delivery.

Public health observatory data and online resources relevant to a scenario area had to meet certain thresholds to provide parity with the other scenario locations. There also had to be sufficient online information for students to be able to conduct thorough analysis of needs and assets within the area, and to identify appropriate community-based interventions.

The allocation of notional learning time within which students were expected to be engaged with the materials varied between providers. For the Australian students learning time was shorter than their UK peers meaning that the experience was more superficial compared to the undergraduate module delivery. However, there was the benefit that Australian instructors could embed a shortened version of the module into their delivery rather than attempting to adopt all the components. This was particularly successful as the students enrolled in the higher-level Australian Graduate Diploma already held skills embedded in the longer delivery approach.

## 6 Conclusion

The collaborative efforts of the institutions participating in this project have identified that final year BSc and postgraduate level environmental health curricula in the UK and Australia hold comparable knowledge, skills, and graduate attribute requirements. Potential for inter-institutional collaboration in learning and teaching is not constrained by accrediting body requirements and their country-specific foci. Environmental health is indeed a global profession with a local focus.

In this situation institutional processes provided the main barriers to collaborative learning. Providers should prioritize timetabling allocations for collaborating modules, to allow the learning space and session times to be matched between providers, with due consideration for time zone differences. Providing learning spaces with suitable AV equipment and ensuring staff are fully trained with that equipment (including problem solving) would improve levels of confidence and willingness to attempt live session sharing. Reducing the timescales, administrative burden, and the uncertainty involved in the processes required to make formal modifications to modules (e.g., assessment changes) would help to support creativity and experimentation in teaching approaches. Formally recognizing successes and taking a lenient position around failures when staff attempt to try new approaches would also embolden those instructors willing to attempt closer teaching relationships with peers in other institutions. At a national/international level closer working between the accrediting institutions and matching of their curricular requirements for recognized programs would facilitate a shared education and subsequent mobility opportunities for environmental practitioners between countries.

There is potential for a common environmental health degree to be developed between the UK and Australia, with both shared and country specific content focusing on local legislation, guidance and culture. Cardiff Metropolitan University already has over 8,000 students on franchise programs duplicating content from existing degree programs but tailoring it for the requirements of partner institutions in different countries. Developing a shared degree program would permit full learning and teaching collaboration, but to date there has not been a willingness to develop a new, shared program between the participating institutions.

To manage international collaborative learning activities between already established programs in higher education institutions, instructors must traverse a series of institutional and practical barriers to allow closer student and staff working. Rather than moving straight to full sharing of learning and teaching, the adoption of a segmented approach to module design allows staged and varied levels of collaboration between institutions, providing a more sustainable environment to facilitate collaboration within these constraining factors and enabling strong relationships to develop between the teaching teams.

This article has described collaboration between providers in affluent western countries with similar legal, political and cultural environments. Teleconferencing facilities enabled live exchange across the teaching spaces and easy communication between participating staff. In less affluent countries asynchronous mechanisms of communication and collaboration can be used and video can be shared though streaming services and file sharing sites, providing a means of managing partnership work where internet access is limited. Where computer, video and projector facilities are unavailable mobile phone applications such as WhatsApp can also be used. Current information technology solutions permit workarounds for most issues of technological capacity found in poorer countries.

The subject area of these interventions requires strong public health observatory data which cannot always be replicated in other nations and has already prevented progress on attempts to extend this collaboration to a Ugandan provider. The nature of environmental health practitioner duties also varies between countries. In this intervention participating countries use environmental health practitioners for public health interventions addressing the wider determinants of health at a community level. PBL is an ideal learning approach for such complex multi-faceted problems but may be employed across other common environmental health areas such as infection control and exposure to environmental pollutants. The approaches used to establish common ground and differing levels of collaboration in this article may be duplicated between other providers, across other subject areas.

PBL provides an appropriate delivery approach to foster the graduate skills and attributes required on environmental health qualifications. Group working and PBL for this module has been seen to exhibit many of the positive aspects noted in the literature including the development of leadership, communication, and independent learning skills. While some of the frustrations associated with PBL and group working were found to have occurred, there are sound foundations of success in this module to build on for future cohorts in the UK, Australia, and further afield.

## Data availability statement

The original contributions presented in the study are included in the article/supplementary materials, further inquiries can be directed to the corresponding author.

## Ethics statement

The studies involving humans were approved by Cardiff Metropolitan University Applied Community Sciences Ethics Panel. The studies were conducted in accordance with the local legislation and institutional requirements. The ethics committee/institutional review board waived the requirement of written informed consent for participation from the participants or the participants’ legal guardians/next of kin because Historical student feedback data was used for this paper. The university had obtained consent to use this for a range of purposes including the module review covered by this paper. Ethical approval was provided for the use of the historical data without a requirement to obtain a separate consent form from the students whose data was included in the study.

## Author contributions

HD: Conceptualization, Investigation, Writing – original draft, Writing – review & editing. GD: Investigation, Writing – original draft, Writing – review & editing. KR: Writing – original draft. MM: Writing – review & editing. AT: Writing – review & editing.

## References

[1] Biggs J Tang C. Teaching for quality learning at university: what the student does. Philadelphia: Open University Press (2011).

[2] Harden RM Stamper N. What is a spiral curriculum? Med Teach. (1999) 21:141–3. doi: 10.1080/01421599979752 21275727

[3] Dahlgren G Whitehead M. The Dahlgren-Whitehead model of health determinants: 30 years on and still chasing rainbows. Public Health. (2021) 199:20–4. doi: 10.1016/j.puhe.2021.08.009, PMID: 34534885

[4] Dawson H. Alami N. H. Bowen K. Maddah D. (2018) ‘The use of virtual reality for public health education with reference to Syrian refugee camps’, in proc. VR/AR in higher education conference 2018. VR/AR in Higher Education Conference 2018, Swansea, Wales: IM Publications, pp. 73–81.

[5] Dyjack D Choonara A Davis G Dawson H Hannelly T Lynch Z . International environmental health skills, knowledge, and qualifications: enhancing professional practice through agreements between countries. J Environ Health. (2023)

[6] Welsh Assembly Government. Credit and qualifications framework for Wales. Cardiff: Welsh Assembly Government (2009).

[7] Quality Assurance Agency. The frameworks for higher education qualifications of UK degree-awarding bodies. Gloucester: Quality Assurance Agency (2014).

[8] Servant-Miklos V. Problem-oriented project work and problem-based learning: “mind the gap!”. Interdiscip J Probl-based Learn. (2020) 14:1–17. doi: 10.14434/ijpbl.v14i1.28596

[9] Moallem M Hung W Dabbagh N. The Wiley handbook of problem-based learning. 1st ed. Newark: Wiley (2019).

[10] Cersosimo G In: Atkinson P Delamont S Cernat A Sakshaug JW Williams RA, editors. Pragmatism. London: SAGE Publications Ltd. (2020)

[11] Moust J Bouhuijs P Scmidt H. Introduction to problem based learning. 4th ed. Groningen: Noordhoff (2021).

[12] Savin-Baden M Major CH. Foundations of problem-based learning 1. Maidenhead: Open University Press (2004).

[13] Muñoz Campos D. Problem-based learning: an experiential strategy for English language teacher education in Chile. Profile Issues Teach Prof Dev. (2017) 19:29–40. doi: 10.15446/profile.v19n1.53310

[14] Griffith E Butler C Csecs J Davis C. An evaluation of a program of problem-based learning within a clinical psychology doctorate. Psychol Teach Rev. (2018) 24:38–54. doi: 10.53841/bpsptr.2018.24.2.38

[15] Li HC Tsai TL. The implementation of problem-based learning in a Taiwanese primary mathematics classroom: lessons learned from the students’ side of the story. Educ Stud. (2017) 43:354–69. doi: 10.1080/03055698.2016.1277138

[16] Dolmans DHJM Loyens SMM Marcq H Gijbels D. Deep and surface learning in problem-based learning: a review of the literature. Adv Health Sci Educ Theory Pract. (2016) 21:1087–112. doi: 10.1007/s10459-015-9645-6, PMID: 26563722 PMC5119847

[17] Balim AG Inel-Ekici D Ozcan E. Concept cartoons supported problem based learning method in middle school science classrooms. J Educ Learn. (2016) 5:272–84. doi: 10.5539/jel.v5n2p272

[18] Trullàs JC Blay C Sarri E Pujol R. Effectiveness of problem-based learning methodology in undergraduate medical education: a scoping review. BMC Med Educ. (2022) 22:104. doi: 10.1186/s12909-022-03154-8, PMID: 35177063 PMC8851721

[19] Shin IS Kim JH. ‘The effect of problem-based learning in nursing education: a meta-analysis. Adv Health Sci Educ Theory Pract. (2013) 18:1103–20. doi: 10.1007/s10459-012-9436-2 23283571

[20] Yew EHJ Goh K. Problem-based learning: an overview of its process and impact on learning. Health Prof Educ. (2016) 2:75–9. doi: 10.1016/j.hpe.2016.01.004

[21] Brown A. Groupwork. 3rd ed. United Kingdom: Routledge (2017).

[22] Jones C Volet S Pino-Pasternak D Heinimäki OP. Interpersonal affect in groupwork: a comparative case study of two small groups with contrasting group dynamics outcomes. Frontline Learn Res. (2022) 10:46–75. doi: 10.14786/flr.v10i1.851

[23] Cartwright NM Patil P Liddle DM Newton G Monk JM. Enhancement of professional Behaviours and perceptions of critical skill job preparedness through the use of a group work contract in fourth-year nutritional science students. Int J High Educ. (2020) 10:27. doi: 10.5430/ijhe.v10n2p27

[24] Hartley P Dawson M Beckingham S. Success in Groupwork. 2nd ed. London: Bloomsbury Publishing Plc (2022).

[25] Poort I Jansen E Hofman A. Does the group matter? Effects of trust, cultural diversity, and group formation on engagement in group work in higher education. High Educ Res Dev. (2022) 41:511–26. doi: 10.1080/07294360.2020.1839024

[26] Sridharan B Tai J Boud D. Does the use of summative peer assessment in collaborative group work inhibit good judgement? High Educ. (2019) 77:853–70. doi: 10.1007/s10734-018-0305-7

[27] Nicol D. Developing the students’ ability to construct feedback. Gloucester: Quality Assurance Agency for Higher Education (2011).

[28] Steensels C Leemans L Buelens H Laga E. Peer assessment: a valuable tool to differentiate between student contributions to group work? Pharm Educ. (2006) 6:111–8. doi: 10.1080/15602210600662279

